# Leukemia cutis as a prominent clinical sign in a dog with acute myeloid leukemia

**DOI:** 10.1111/vcp.13382

**Published:** 2024-09-17

**Authors:** Francesco Rogato, Balazs Szladovits, Henny Martineau, Andrew D. Yale, Jordan L. Mitchell, Emma J. Holmes, Rachel H. Gardner, Alexandra Guillén

**Affiliations:** ^1^ Department of Clinical Sciences and Services Royal Veterinary College Hatfield Hertfordshire UK; ^2^ Department of Pathobiology and Population Sciences Royal Veterinary College Hatfield Hertfordshire UK

**Keywords:** bone marrow, canine, flow‐cytometry, immunohistochemistry, lymphadenopathy, skin

## Abstract

An eight‐year‐old male neutered crossbreed dog presented with erosive and ulcerative cutaneous lesions in the inguinal regions, the medial aspect of both thighs, and the stifles. Hematologic assessment revealed nonregenerative anemia, thrombocytopenia, and high numbers of neoplastic mononuclear cells with a variable degree of maturation. The mononuclear neoplastic cells, with nuclei measuring 10–20 microns in diameter, accounted for 57% of the nucleated blood cells. In addition, the blood contained increased numbers of mature neutrophils and monocytes with atypical morphology. Cytologic examination of the right popliteal lymph node found high numbers of large mononuclear cells with similar morphology to those in the peripheral blood. Flow cytometry of peripheral blood revealed expression by the mononuclear neoplastic cells of the pan‐leukocyte marker CD45 and myeloid markers CD14, MAC387, and myeloperoxidase (MPO). These results confirmed a diagnosis of acute myeloid leukemia (AML). Computed tomography found moderate nodular hepatosplenomegaly and multifocal bi‐cavitary lymphadenopathy. Histopathologic examination of biopsies from the cutaneous lesions identified infiltration of the dermis by intermediate to large neoplastic round cells. Further treatment was declined, and the owners elected euthanasia. Postmortem examination confirmed AML involvement in the bone marrow, peripheral and intracavitary lymph nodes, heart, liver, kidney, and skin. Neoplastic cells in the bone marrow and skin showed positive immunolabeling for ionized calcium‐binding adaptor protein 1 and MPO. To the best of our knowledge, this is the first report of ulcerative cutaneous lesions observed among the presenting clinical signs in a dog with AML and secondary leukemia cutis.

## 1 CASE PRESENTATION

**FIGURE 1:**
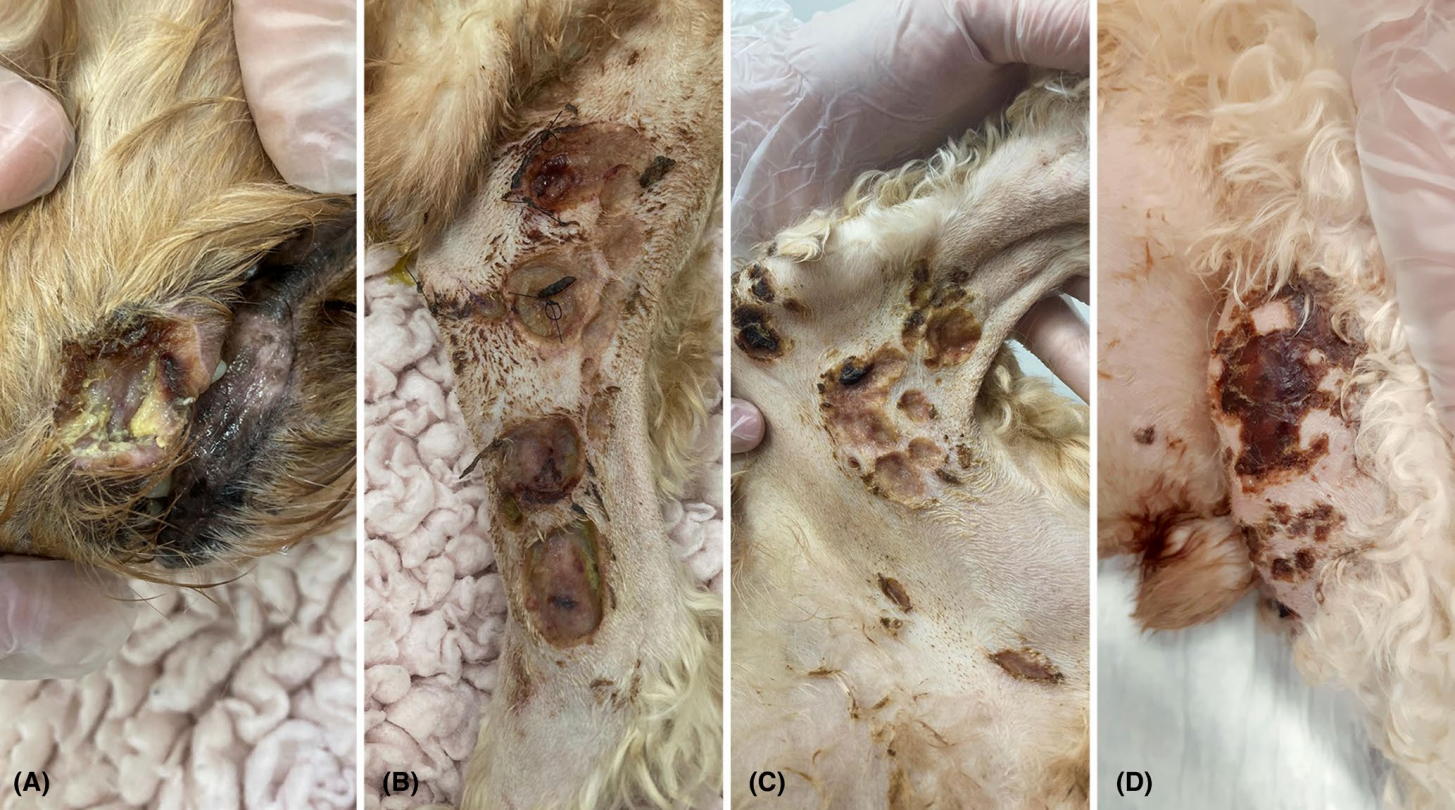
Sites of ulcerative cutaneous lesions from a dog with acute myeloid leukemia (AML). (A) A focal necrotic ulcer on the rostral aspect of the left maxillary lip at the mucocutaneous junction in a dog with cutaneous involvement of AML. Additional multiple, marked, multifocal to coalescing, circular to irregular areas of cutaneous erosion and ulceration were present over the left inguinal region and medial hindlimb region (B), right medial hindlimb (C), and left craniolateral stifle (D).

An eight‐year‐old male neutered crossbreed dog was referred for progressive lethargy of two weeks duration nonresponsive to oral meloxicam and rapid development of multiple, coalescing, erosive, and ulcerative cutaneous lesions over the two days prior to referral. On physical examination, the dog had mild tachycardia (140 beats per minute), cranial abdominal organomegaly, moderate bilateral popliteal lymphadenomegaly, and pyrexia (39.6°C). There were multiple (20–30), multifocal to coalescing, circular to irregular areas of cutaneous erosion and ulceration over the ventral abdomen and inguinal region, extending distally over the craniomedial aspect of both hind limbs. A focal ulcer on the left maxillary lip and an erosion on the right maxillary oral mucosa were also seen (Figure 1).

**FIGURE 2:**
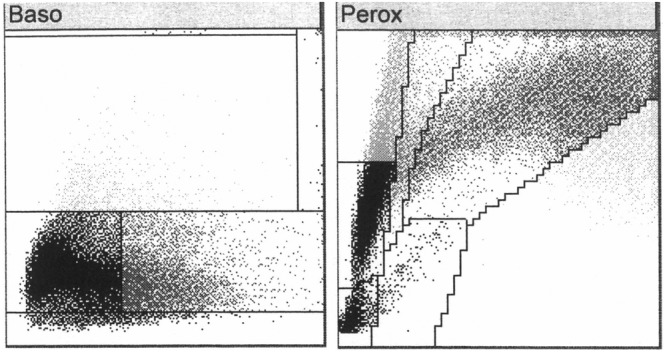
Cytograms of the ADVIA 2120i of a dog with acute myeloid leukemia ML. The basophil channel has a marked extension of the mononuclear cloud into the lysis‐resistant portion of the graph, typical for blast cells. The peroxidase channel has a prominent signal for large unstained cells and the merging of the monocyte and neutrophil clouds, which extend widely into the strongly peroxidase‐positive region of the graph.

**FIGURE 3:**
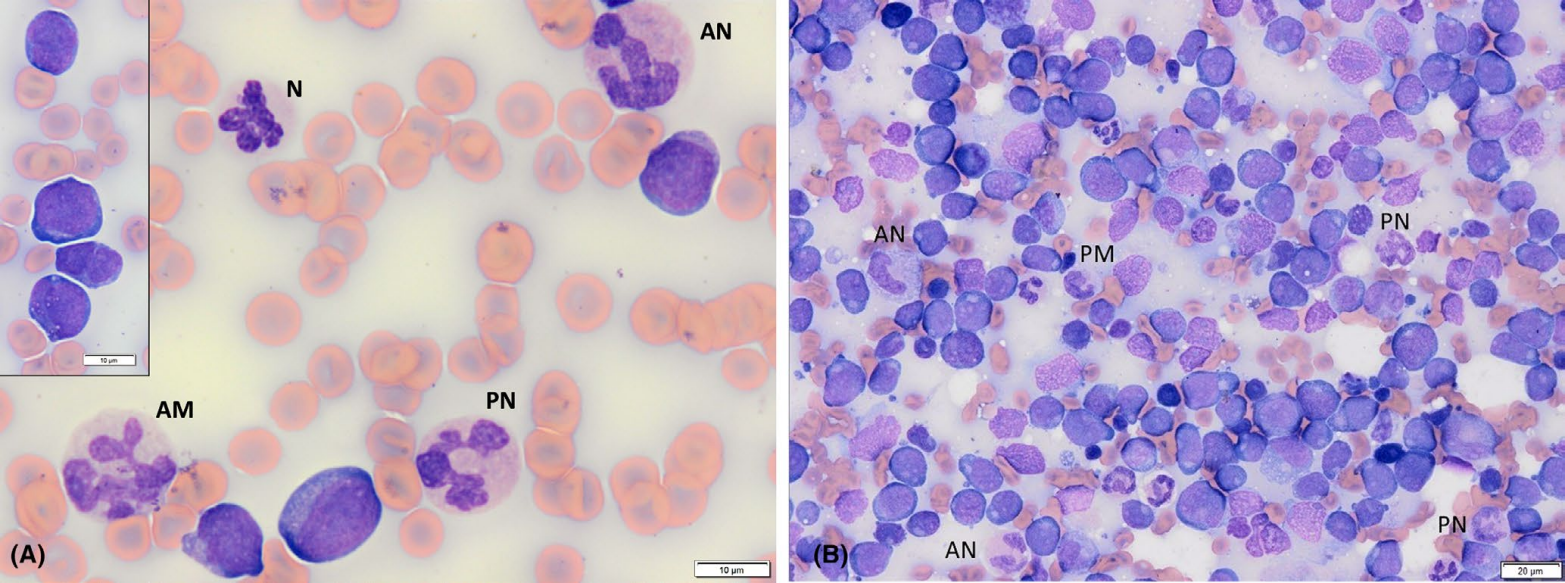
(A) A mixture of neoplastic immature cells and atypical mature cells in the peripheral blood of a dog with acute myeloid leukemia (AML). Abnormally segmented, giant neutrophils with orange‐pink staining cytoplasm and hypersegmented monocytes were common, with occasional cells bridging in appearance. N: Neutrophil, AN: Atypical neutrophil, AM: Atypical monocyte, PN: Possible neutrophil. INSET: Immature neoplastic cells with nuclear morphology varying from round to cleaved to scalloped to lobed. Modified Wright's stain, 100x objective. (B) Right popliteal lymph node aspirate of a dog with AML predominated by high numbers of neoplastic cells, with a similar range of morphologies as seen in the peripheral blood. Low numbers of atypical neutrophils, monocytes, and cells with bridging morphologies are also present, further matching the blood smear findings. AN: Atypical neutrophil, PN: Possible neutrophil, PM: Possible monocyte. Modified Wright's stain, 40x objective.

CBC and blood film examination identified a moderate, nonregenerative, normocytic, normochromic anemia, moderate thrombocytopenia, marked leukocytosis, mild neutrophilia, and moderate monocytosis, with the majority of leukocytes on manual differential classified as “Other” (Table 1). Cytograms of the basophil and peroxidase channels are present in Figure 2. The unknown mononuclear cells had round to indented to floriform to lobed nuclei measuring 10–20 microns, finely granular chromatin, single to multiple small nucleoli (up to 4), and a scant to small amount of basophilic cytoplasm with low numbers of punctate vacuoles (Figure 3A). Many cells were classified as monocytes with hypersegmented nuclei and pale blue‐gray cytoplasm with occasional vacuoles. High proportions of neutrophils had markedly abnormal features, including frequent giant cell size (up to 20 μm), loosely clumped to coarse chromatin, irregular segmentation (side‐branching, hypersegmentation, and thin strands between large lobes), and orange‐pink staining cytoplasm. Occasional cells had prominent cytoplasmic foaminess and diffuse basophilia. Overlap between neutrophil and monocyte morphologies occasionally made differentiation difficult. The morphology and high concentration of circulating immature cells (>20%) were strongly suggestive of acute myeloid leukemia (AML).^1^, ^2^ Although the prominent atypia of the neutrophils and monocytes raised concern for myelomonocytic AML (AML‐M4), a monocytic lineage AML (AML‐M5b) and lymphoid origin with secondary dysmyelopoiesis could not be ruled out. The nonregenerative anemia and thrombocytopenia were suspected to be related to myelophthisis; thus, the lack of neutropenia and atypical appearance of the neutrophils further supported the neutrophilic line's involvement in the neoplastic process (M4 vs. M5). Concurrent immune‐mediated destruction of erythrocytes and thrombocytes or immune‐mediated (secondary) destruction of the erythroid line with consumption of platelets could not be excluded completely.

**TABLE 1:** Hematologic data from a dog with acute myeloid leukemia.

Test	Result	Reference interval	Units
WBC	81.6*	6.0–17.1	10^9^/L
Segmented Neutrophils	16.3*	3.0–11.5	10^9^/L
Segmented Neutrophils %	20		%
Lymphocytes	4.9*	1.0–4.8	10^9^/L
Lymphocytes %	6		%
Monocytes	13.9*	0.1–1.5	10^9^/L
Monocytes %	17		%
Eosinophils	0.0	0–1.3	10^9^/L
Eosinophils %	0		%
Basophils	0.0	0–0	10^9^/L
Basophils %	0		%
OTHER	46.5*	0	10^9^/L
OTHER %	57		%
RBC	2.28*	5.50–8.50	10^12^/L
HGB	53*	120–180	g/L
HCT	16.3*	37.0–55.0	%
MCV	71.2	60.0–77.0	fL
MCH	23.4	19.5–24.5	pg
MCHC	329	320–360	g/L
RDW	17.4		%
Reticulocytes	13.68		10^9^/L
PLT	36* 90^b^	150–900	10^9^/L
Platelets	No clumps; decreased		
PCV	18		
In saline agglutination	Negative		
Plasma color	Mild icterus		

*Note*: Reference intervals validated at Diagnostic Laboratory Services, Royal Veterinary College, United Kingdom. Cell concentrations and red blood cell indices were measured with an ADVIA 2120i automated hematologic analyzer (Siemens), and a board‐certified clinical pathologist performed manual leukocyte differential on blood smears. The “*” is used to highlight the results that are outside the reference interval.

Abbreviations: HCT, hematocrit; HGB, hemoglobin concentration; MCH, mean cell hemoglobin; MCHC, mean cell hemoglobin concentration; MCV, mean cell volume; PCV, packed cell volume; PLT, platelet concentration; RDW, red cell distribution width.

^b^ Smear estimate.

Serum biochemical analysis revealed moderate to marked hypoalbuminemia (16.9 g/L; RI, 26.3–38.2 g/L), mild hyperglobulinemia (51.2 g/L; RI, 23.4–42.2 g/L), and markedly increased C‐reactive protein (108.6 mg/L; RI, 0–10.0 mg/L; Gentian assay, Beckman Coulter AU680), which were indicative of ongoing inflammation, while dermatopathy could have also contributed to the severity of the hypoalbuminemia.

Cytologic examination of the right popliteal lymph node found a heterogeneous population of predominantly medium to large immature round cells (>75%) with morphologic features similar to those observed in peripheral blood (Figure 3B). A low proportion of these cells appeared more myeloid or monocytoid with reniform to bilobed nuclei and had an increased amount of amphophilic cytoplasm, and rare cells contained low numbers of magenta granules. Occasional mitotic figures were seen. Furthermore, low numbers of atypical neutrophils and monocytes, similar to those seen in peripheral blood, were observed. The proportion of these cells exceeded that expected from blood contamination. Only low numbers of small lymphocytes (approx. 10%) were present, and occasional erythrophages were infiltrating the lymph node. While the high numbers of immature cells would be suggestive of stage V lymphoma or lymphoid leukemia, the variability of nuclear morphology, along with the atypical neutrophils and monocytes, was highly suspicious of myeloid leukemia infiltrating the lymph node. Computed tomography of the thorax and abdomen identified moderate nodular hepatosplenomegaly and multifocal bicavitary lymphadenopathy. Wedge‐shaped, nonenhancing splenic lesions extending from one side of the capsule, consistent with infarcts, were also observed. Fine‐acute myeloid leukemia needle aspirates of visceral organs were not attempted due to the thrombocytopenia.

Flow cytometry was performed on peripheral blood. Cells were stained with a panel of antibodies as shown in Table 2.

**TABLE 2:** Antibodies clones and methodology used for the flow cytometry in a dog with acute myeloid leukemia.

Antibody target	Clone	Isotype	Working dilution	Suggested bolume (μL)	End volume (μL)	Negative control	Material name	Isotype	Working dilution	Suggested volume (μL)	End volume (μL)
CD3	CA17.2A12	mlgG1	1 in 4	50:150	200	N05F	MCA6005F	rlgG2a	1 in 10	20:180	200
CD4	YKIX302.9	rlgG2a	1 in 8	16:144	160	N06F	MCA6006F	rlgG2b	1 in 10	20:180	200
CD5	YKIX322.3	rlgG2a	1 in 10	20:180	200	N11F	MCA1211F	rlgG1	1 in 40	10:190	200
CD8	YCATE55.9	rlgG1	1 in 4	50:150	200	N12PE	MCA1212PE	rlG2a	1 in 10	20:180	200
CD14	TÜK4	mlgG2a	1 in 16	10:150	160	N12F	MCA1212F	rlgG2a	1 in 10	20:180	200
CD21	CA2.1D6	mlgG1	Neat			N28PE	MCA928PE	mlgG1	1 in 5	40:160	200
CD34	1H6	mlgG1	1 in 10	20:180	200	N28F	MCA928F	mlgG1	1 in 5	40:160	200
CD45	YKIX716.13	rIgG2b	1 in 10	20:180	200	N29F	MCA929F	mlgG2a	1 in 10	10:90	100
CD79a	JCB117	mlgG1	1 in 4	50:150	200						
MPO	2C7	mlgG1	1 in 10	10:90	100						
MAC387	MAC387	mlgG1	1 in 5	40:160	100						
MHCII	YKIX334.2	rlgG2a	1 in 5	40:160	200						

*Note*: The flow cytometry was performed by DWR Diagnostic Laboratory, United Kingdom. The antibody clone CD79a was purchased from Agilent Technologies. All the remaining assessed antibody clones were purchased from Bio‐Rad. Aliquots of blood containing 1 × 10^6^ cells were placed in Eppendorf tubes. Red cells were lysed by adding 1 mL of red cell lysis buffer (Pharmalyse, Becton Dickinson) to each tube and incubating for 15 min at room temperature, followed by washing in phosphate‐buffered saline. Cells were then incubated for 30 min with the conjugated antibodies, then washed in phosphate‐buffered saline, and resuspended. CD79a, MPO, and MAC387 antibodies detect antigens on the inner surface of the cell membrane, so cells were first fixed in a formaldehyde‐based fixative for 15 min, washed, and then incubated with a permeabilization reagent together with the conjugated antibody for 30 min using a commercially available permeabilization kit (Leucoperm, Bio‐Rad). The stained cells were washed and then placed on a 96‐well plate for analysis. Propidium iodide was used to distinguish live and dead cells (it is taken up by dead cells) and so was added to wells containing nonpermeabilized cells immediately prior to analysis, but not to the permeabilized cells since it would bind to all fixed/permeabilized cells.

**FIGURE 4:**
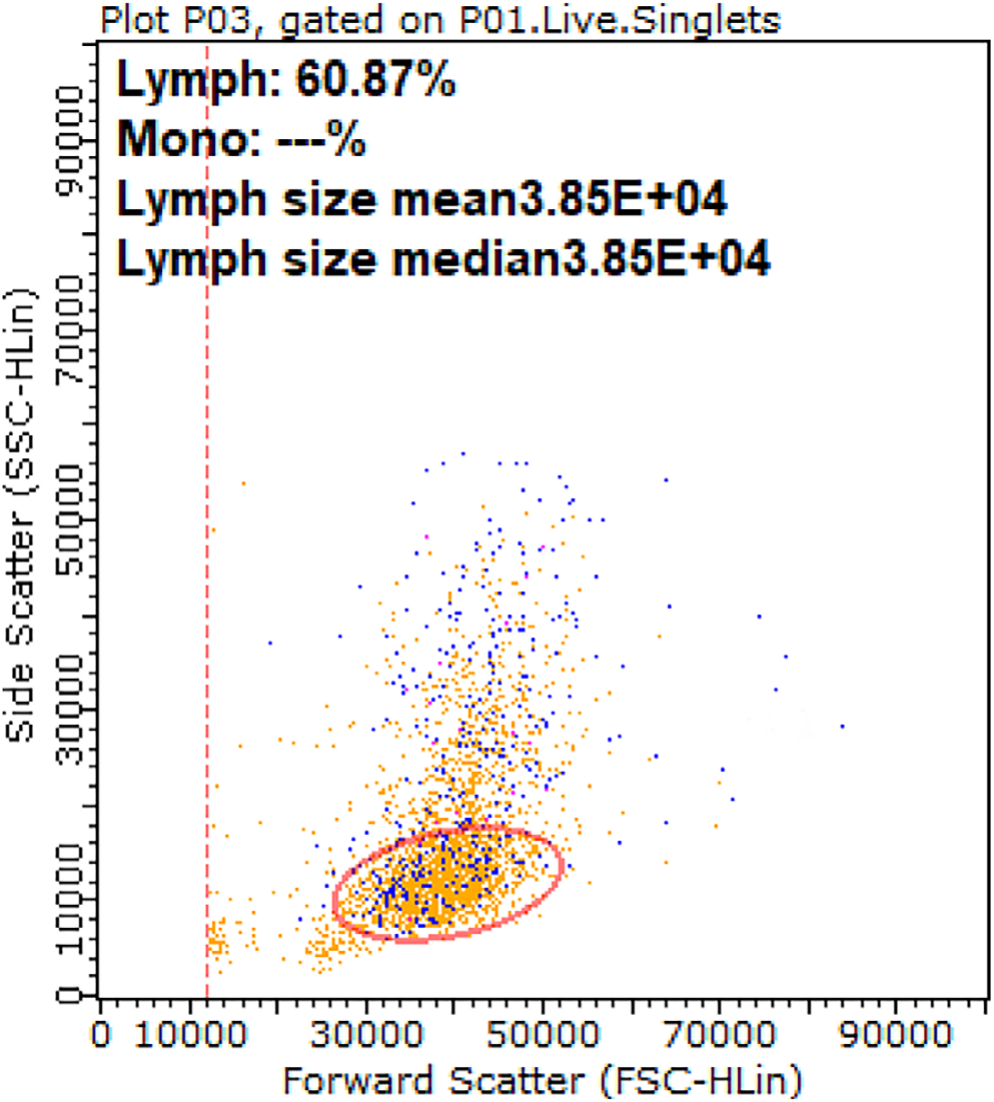
Side scatter versus forward scatter plot highlighting gating of the neoplastic large mononuclear population on flow cytometry of a dog with acute myeloid leukemia. Neoplastic cells are incorrectly labeled as lymphocytes by the software. Side scatter indicates the internal complexity/granularity, and forward scatter indicates the relative size of the cells.

For flow cytometric analysis, dead cells (staining with PI) were excluded, and single cells that have the same width and height were selected. The large mononuclear cells were identified on the side versus forward scatter plot and gated, excluding neutrophils and small lymphocytes (Figure 4). Flow cytometry on peripheral blood identified that the majority of the neoplastic large cells expressed the pan‐leukocyte marker CD45, and a small proportion expressed myeloid markers (Table 3). These cells did not express lymphoid markers (CD3, CD4, CD5, CD8, CD21, CD79a, and major histocompatibility complex MHC‐II). In summary, the cellular morphology determined on blood smear examination, together with the percentage of circulating immature cells (≥20%) from the CBC differential and the immunophenotype of the neoplastic cells (>3.0% CD14+MHCII cells) documented on flow cytometry, supported the diagnosis of AML, with AML‐M4 considered more likely.^3^, ^4^

**TABLE 3:** Flow cytometry results from a dog with acute myeloid leukemia.

Antigen	Cell type	Result
CD3	T cells	Negative
CD5	T cells, subset of B cells	Negative
CD4	T helper cells, neutrophils, activated monocytes	Negative
CD8	Cytotoxic T cells	Negative
CD21	B cells	Negative
CD79a	B cells	Negative
MHC II	Lymphocytes, monocytes	Negative
CD45	All leukocytes	92% positive
MPO	Myeloid cells	10% positive
MAC387	Myeloid cells	19% positive
CD14	Monocytes	19% positive
CD34	Stem cells and early committed myeloid and lymphoid progenitors	Negative

Abbreviations: MAC387, calprotectin; MHC II, major histocompatibility complex II; MPO, myeloperoxidase.

Bacterial culture from the cutaneous lesions isolated a profuse mixed growth of *Streptococcus canis* and *Staphylococcus pseudintermedius*. Histopathologic examination of the skin (three 8 mm punch biopsies were obtained from the medial aspect of the right thigh) revealed effacement of dermal architecture by sheets of intermediate to large neoplastic round cells with marked pleomorphism and multifocal extensive necrosis with myriads of bacterial organisms and poor cell preservation. Neoplastic cells had well‐defined borders, a small amount of eosinophilic cytoplasm, and central, hyperchromatic, round to reniform to indented nuclei with considerable anisokaryosis. The dermis and subcutis were diffusely and markedly expanded by edema, and large inflammatory cell infiltrates of predominantly degenerate and viable neutrophils, lymphocytes, and plasma cells were also present. Admixed with the inflammatory cell infiltrates were large multifocal colonies of basophilic coccoid bacteria, as well as degenerate neutrophils and neoplastic cells. Neoplastic cells were also present in dermal vasculature. The epidermis was ulcerated and overlain by necrotic debris and eosinophilic fibrin and surrounded by degenerate and viable neutrophils. The presence of neoplastic cells raised concern for cutaneous involvement, that is, leukemia cutis (LC).

Due to the extent of the lesions and degree of tissue necrosis, surgical debridement and open wound management were recommended. However, as the overall prognosis was poor, the owner declined chemotherapy, and euthanasia was elected. Gross postmortem examination confirmed multifocal to coalescing areas of erosion and ulceration over the ventral abdomen, inguinal region, hindlimbs, lips, and oral mucosa. The liver was enlarged. It was light brown with rounded lobe edges and had a friable texture. It weighed 852 g, which was 5.7% of body weight (3–3.5% of body weight is considered normal). Along the length of the spleen, there were multifocal, raised, dark red nodules, the largest of which measured 3 cm x 1 cm x 0.5 cm. The cut surface of this nodule was dark red with a central white focus that extended into the splenic parenchyma. On the surface of both kidneys were multifocal, white, circular, flat areas ranging in size from 3 mm to 9 mm diameter. Examination of bone marrow at the level of the left femoral metaphysis found it to be red and soft with a central off‐white area and granular texture. Peripheral moderate lymphadenomegaly was noted.

**FIGURE 5:**
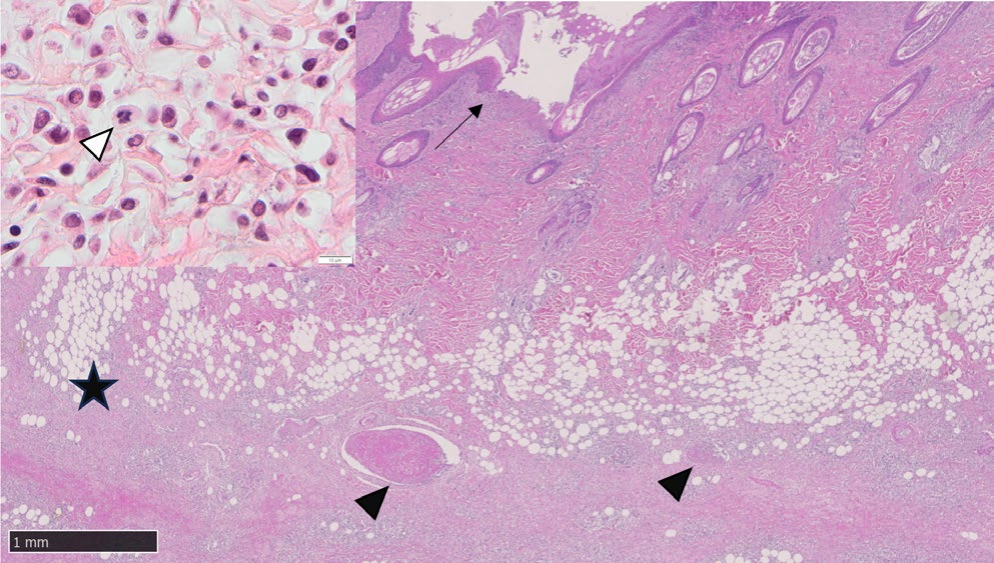
Whole‐slide scanned image (WSI) and photomicrograph of a postmortem sample of the skin from the right medial thigh of a dog with acute myeloid leukemia (AML). Within the dermis and subcutis are multifocal to coalescing infiltrates of moderate numbers of neoplastic round cells (black star). There are intravascular fibrin thrombi within the subcutaneous vasculature (black arrowhead), and the overlying epidermis is focally ulcerated (black arrow) and overlain by fibrin, necrotic debris, viable and degenerate neutrophils, and coccoid bacteria. H&E, ×0.8 magnification. (Inset) Higher magnification of the pleomorphic neoplastic round cell dermal and subcutaneous infiltrate, including bizarre mitoses (white arrowhead). H&E, ×100 objective.

**FIGURE 6:**
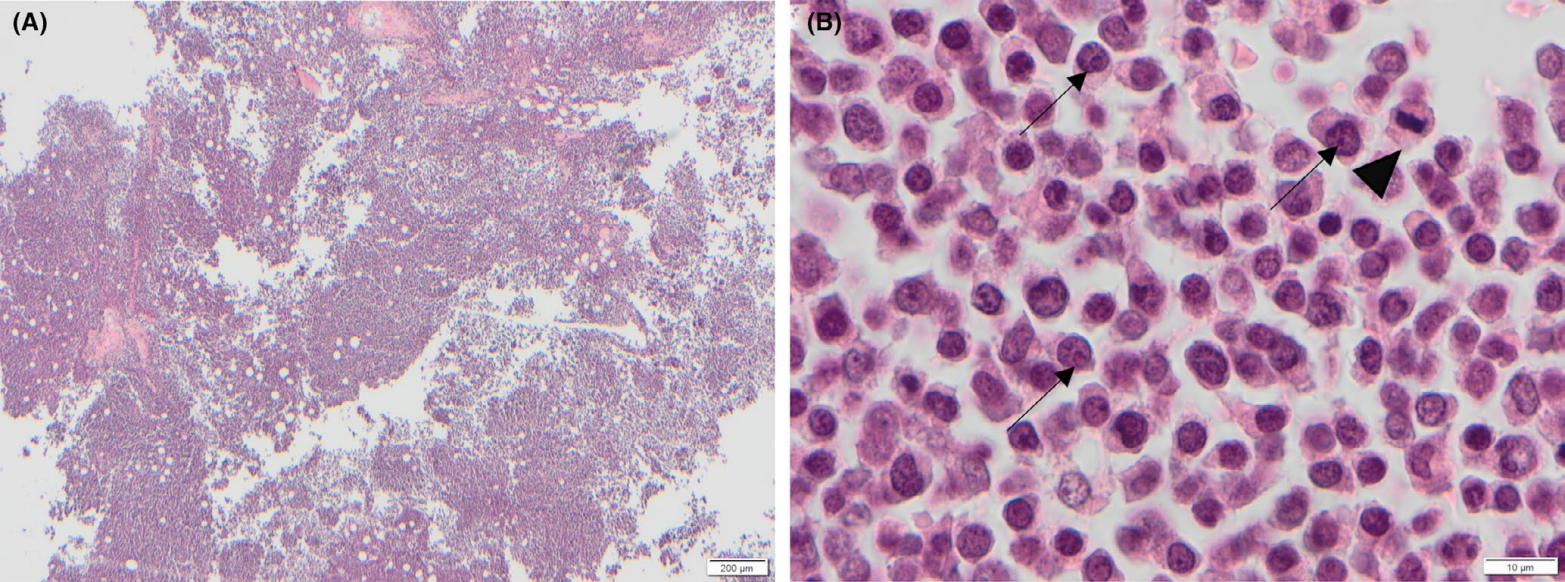
Photomicrographs of the left femoral bone marrow, obtained at postmortem examination, from a dog with acute myeloid leukemia. (A) The bone marrow is markedly hypercellular and densely cellular with low numbers of scattered adipocytes. H&E, ×4 objective. (B) The neoplastic round cells exhibit moderate anisokaryosis, with prominent nucleoli (arrows) and frequent mitoses (arrowhead). H&E, ×100 objective.

**FIGURE 7:**
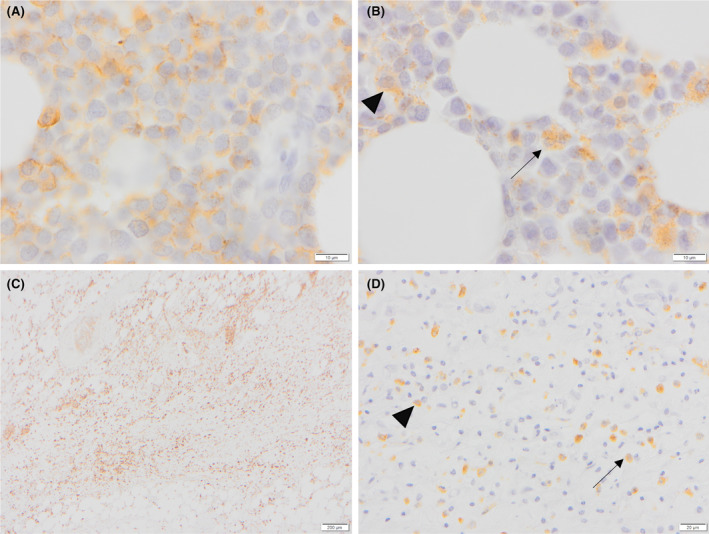
Photomicrographs of immunohistochemistry of the femoral bone marrow and skin from the left medial thigh of a dog with acute myeloid leukemia. (A) Bone marrow. Large numbers of round cells have positive cytoplasmic, finely stippled immunolabeling for ionized calcium‐binding adaptor protein 1 ba‐1). IHC, ×100 objective. (B) Bone marrow. Smaller numbers of neoplastic round cells have positive, cytoplasmic, granular immunolabeling for myeloperoxidase (MPO). Neoplastic cells have reniform (arrowhead) to bilobed (arrow) nuclei. IHC, ×20. (C) Haired skin. Marked numbers of dermal and subcutaneous infiltrating neoplastic cells have positive immunolabeling for Iba1. IHC, ×4 objective. (D) Haired skin. In the subcutis, there are scattered round cells with round‐to‐reniform (arrowhead) to bilobed (arrow) nuclei and positive cytoplasmic, granular immunolabeling for MPO. IHC, ×20 objective.

Microscopic examination of the ulcerated skin from the left and right medial thighs and ventral abdomen identified neoplastic cells in the dermis and subcutis together with multifocal intravascular fibrin thrombi (Figure 5). Microscopic examination of the bone marrow was significantly hindered due to postmortem autolysis. Within this limitation, it was markedly hypercellular and densely cellular, with neoplastic cells forming closely packed sheets, interspersed by adipocytes, with scant stroma (Figure 6A). Cells were round to polygonal and variable in size, with indistinct borders, a scant to mild amount of eosinophilic to intensely basophilic, occasionally faintly granular cytoplasm, and a round to reniform, central to eccentric hyperchromatic nucleus and finely stippled to clumped chromatin. There was moderate anisokaryosis and anisocytosis. Mitoses were 47 in 10 HPF (x 400 objective; 2.37mm^2^), with occasional bizarre mitoses (Figure 6B). Neoplastic round cells were also identified in the right popliteal lymph node, tracheobronchial lymph node, mesenteric lymph node, heart, liver, and kidney. Immunohistochemistry was performed on sections of ulcerated skin from the left and right medial thighs, ventral abdomen, and femoral bone marrow using antibodies raised against ionized calcium‐binding adaptor protein 1 (Iba‐1, Wako (Alpha labs) 09–19 741, polyclonal, AR HIER pH 6 Sodium Citrate buffer, Ab dilution 1:3000), MPO (Dako/Agilent A0398, polyclonal, antigen retrieval (AR) Proteinase K 5 mins, Ab dilution 1:3000), CD3 (Dako A0452, polyclonal, AR HIER pH 6 Sodium Citrate buffer, Ab dilution 1:100), CD20 (Thermo Fisher PA5‐16701, polyclonal, AR HIER pH 6 Sodium Citrate buffer, Ab dilution 1:600), and CD79a (BioRad MCA2538GA, monoclonal clone HM57, AR HIER pH 6 Sodium Citrate buffer, Ab dilution 1:2000). In both locations, cytoplasmic immunolabeling for Iba‐1 was present in approximately 70% of neoplastic cells, and a smaller proportion (20%) labeled positively for MPO (Figure 7A–D). The cytologic, histologic, immunophenotypic, and immunohistochemical findings were most compatible with AML‐M4 and LC.

## 2 DISCUSSION

This report describes a novel case of AML in a dog, where ulcerative cutaneous lesions consisting of neoplastic myeloid cell infiltrate (LC) were among the initial clinical signs observed. Leukemia cutis is considered rare in dogs, with no skin changes reported among the presenting clinical signs or physical examination findings for AML in the larger case series available literature.^5^, ^6^, ^7^ There is a case report of LC in a dog with T‐cell chronic lymphocytic leukemia (CLL).^8^ In this dog, skin lesions developed approximately one year after the initial diagnosis of leukemia, which may be related to the chronic and slow‐progressive nature of the underlying neoplastic disease, and the lesions had a different appearance from the present case report, being characterized by bilateral multifocal purpura and ecchymoses. There is, however, some overlap in lesion distribution, as lesions were found in the inguinal regions and over the thighs in both cases.^8^ Myeloid cells were speculated to cause a cutaneous neoplasm in a dog, as described by Gianella et al.^9^ That dog was affected by chronic and progressive weakness accompanied by two cutaneous lesions of more rapid onset. Although ulcerated, the lesions of the dog reported appeared 2 weeks prior to referral as single raised firm nodules in two different locations (left thoracic and left abdominal wall), rendering the clinical presentation different from the one described in the present case report. Histopathologic assessment of the cutaneous lesions revealed infiltrates of neoplastic round cells in the dermis extending into the subcutis, similar to our case. However, the presence of neoplastic cells in the peripheral circulation and bone marrow was ruled out by normal CBC and bone marrow findings. Infiltrates of neoplastic cells were subsequently confirmed at postmortem in visceral organs such as the liver, kidneys, and lungs. Extensive immunocytochemical and immunohistochemical profiles supported myeloid or possibly NK origin for the neoplastic cells. In people, skin infiltration may precede bone marrow and peripheral blood infiltration in a minority of patients with leukemia (approximately <10%), and extramedullary tumors of immature granulocytic cells causing tumor masses, described as “myeloid sarcomas” or “granulocytic sarcomas,” may very rarely present in isolation (in aleukemic patients or prior to the development of the leukemia in the bone marrow and peripheral circulation).^10^ Alternatively, the dog described by Gianella et al. could represent either primary visceral neoplasia with involvement of the skin or primary cutaneous neoplasia with involvement of the visceral organs.

Although considered rare in dogs, AML is the most commonly diagnosed acute leukemia, which is similar to the human literature.^11^, ^12^, ^13^, ^14^ Clinical signs are often nonspecific (eg, inappetence, lethargy), and physical examination findings can vary greatly and include fever, tachycardia, and peripheral lymphadenomegaly.^5^, ^6^

Leukemia cutis is more commonly seen in people with AML with monocytic or myelomonocytic morphology (M4 and M5), although incidences may vary in different countries.^15^, ^16^ Cutaneous manifestations of leukemia are variable in people and traditionally divided into specific and nonspecific skin changes based on clinical and histopathological criteria.^17^ Specific skin changes include all the lesions characterized by neoplastic cutaneous infiltration (generally classified as LC), while nonspecific skin changes are usually the result of insufficient hematopoiesis (eg, thrombocytopenia may cause thrombocytopenic purpura) or expression of a cutaneous paraneoplastic disorder (eg, pyoderma gangrenosum).^17^ These usually develop after the diagnosis of leukemia, and LC lesions include papules, maculae, nodules, plaques, and ulcers, with no site predilection.^15^, ^16^ The presence of neoplastic cells in the dermis and subcutis documented in our case renders the lesions “specific” and, therefore, is classified as LC. Ulcers can occasionally appear as multiple lesions of a few centimeters in diameter and may also affect unusual sites such as the groin, scrotum, and face.^15^, ^16^ The pathogenesis involves interaction between different cytokine receptors and adhesion molecules responsible for the migration and “colonization” of neoplastic cells to specific tissue.^18^ The development of the cutaneous lesions and, more specifically, ulcers could also be attributed to leukostasis and disruption of normal blood flow by aggregates and thrombi of leukemic cells in the capillaries and small blood vessels, resulting in hemorrhage and necrosis in the affected areas as previously reported in people and horses.^19^, ^20^ This process could have been a contributing factor to the development of the cutaneous lesions, also in our case. In fact, although the diffuse infiltration of Iba‐1 and MPO‐positive neoplastic cells in the dermis and subcutis was not predominantly localized to areas of ulceration, the infiltration by the neoplastic cells and/or intravascular fibrin thrombi had likely caused the subsequent ischemia and necrosis, which may explain the rapid development and appearance of the lesions.

The disrupted blood flow causing the ischemia and necrosis, together with a reduced immune response by the neoplastic cells, likely favored the onset of secondary bacterial overgrowth in the cutaneous lesions. These lesions were mostly localized in the anatomical areas of higher moisture, therefore providing optimal conditions for bacteria colonization.

The clinical morphology and distribution of cutaneous lesions are not pathognomonic for LC. Davis et al. showed overlapping clinical and cytological features between AML and lymphoid neoplasia in dogs, highlighting the need for cytochemical staining and immunophenotyping to reach a definitive diagnosis.^6^ In our case, acute lymphoid leukemia and stage V lymphoma were considered differentials, but the nuclear morphology and progression toward atypical monocytes and neutrophils made these less likely. Flow cytometry on peripheral blood revealed expression of myeloid markers (MPO, MAC387, and CD14) and absence of expression of lymphoid markers, further supporting myeloid origin.^4^ The precursor marker CD34 is inconsistently expressed by blast cells; therefore, the absence of expression in our case did not exclude a diagnosis of acute leukemia.^21^

The myeloid origin of the neoplastic cells was further supported by immunohistochemistry performed on postmortem samples from the bone marrow and skin. Neoplastic cells were positive for monocytic markers (MPO, Iba‐1) and negative for lymphoid markers (CD3, CD20, and CD79), excluding lymphoma as a differential diagnosis. Myeloperoxidase and Iba‐1 expression confirmed the myeloid origin of the neoplastic cells.^22^, ^23^

A classification system for AML in dogs was developed following the adaptation of the French‐American‐British (FAB) criteria for the classification of AML in people, but more recently, some authors have adopted the World Health Organization (WHO) classification system.^1^, ^2^ Notably, a cutoff of >20% rather than 30% myeloid blasts in the blood or bone marrow for diagnosis of AML is used, although this would not affect the diagnosis in this case. Detailed classification of the different leukemia subtypes has only been possible due to the development of cytochemical staining and immunophenotyping by flow cytometry.^1^, ^5^, ^6^, ^7^, ^24^ Both in dogs and people, myelomonocytic leukemia (M4) and monocytic leukemia (M5a, M5b) are the most frequently reported types of AML.^6^, ^14^ In addition, specific mutations, epigenetic aberrations, and molecular lesions found to be prognostically and therapeutically relevant have been identified in FLT3, C‐KIT, and RAS oncogenes in canine leukemias, suggesting common oncopathogenic pathways across species.^11^

Treatment of underlying leukemia in people usually results in partial to complete resolution of the LC lesions with no significant differences in recurrence‐free survival and average duration of complete remission compared to patients without LC.^25^ In dogs, clinical progression of AML is usually rapid, and although prognosis is improved with chemotherapy, it remains poor, with median survival times rarely exceeding two months.^5^, ^6^

In summary, this report describes the diagnostic challenges and cytologic and histopathologic features associated with an unusual presentation of AML in a dog. To the authors' knowledge, this is the first report of ulcerative cutaneous lesions observed among the presenting clinical signs in a dog with AML and secondary leukemia cutis.

## FUNDING INFORMATION

The authors received no financial support for the research, authorship, and/or publication of this article.

## CONFLICT OF INTEREST STATEMENT

The authors declare no potential conflicts of interest with respect to the research, authorship, and/or publication of this article.

## References

[1] Jain NC , Blue JT , Grindem CB , et al. Proposed criteria for classification of acute myeloid leukemia in dogs and cats. Vet Clin Pathol. 1991;20(3):63‐82. doi:10.1111/j.1939-165x.1991.tb00571.x 12673541

[2] Swerdlow SH , Campo E , Harris NL , et al., eds. WHO classification of Tumours of Haematopoietic and lymphoid tissues. International Agency for Research on Cancer; 2017.

[3] Harris RA , Rout ED , Yoshimoto JA , Avery PR , Avery AC . Using digital RNA counting to establish flow cytometry diagnostic criteria for subtypes of CD34+ canine acute leukaemia. Vet Comp Oncol. 2022;20(3):710‐719. doi:10.1111/vco.12825 35491468 PMC9544023

[4] Villiers E , Baines S , Law AM , Mallows V . Identification of acute myeloid leukemia in dogs using flow cytometry with myeloperoxidase, MAC387, and a canine neutrophil‐specific antibody. Vet Clin Pathol. 2006;35(1):55‐71. doi:10.1111/j.1939-165x.2006.tb00089.x 16511792

[5] Bennett AL , Williams LE , Ferguson MW , et al. Canine acute leukaemia: 50 cases (1989‐2014). Vet Comp Oncol. 2017;15(3):1101‐1114. doi:10.1111/vco.12251 27402031 PMC5233675

[6] Davis LL , Hume KR , Stokol T . A retrospective review of acute myeloid leukaemia in 35 dogs diagnosed by a combination of morphologic findings, flow cytometric immunophenotyping and cytochemical staining results (2007‐2015). Vet Comp Oncol. 2018;16(2):268‐275. doi:10.1111/vco.12377 29239119

[7] Adam F , Villiers E , Watson S , Coyne K , Blackwood L . Clinical pathological and epidemiological assessment of morphologically and immunologically confirmed canine leukaemia. Vet Comp Oncol. 2009;7(3):181‐195. doi:10.1111/j.1476-5829.2009.00189.x 19691647

[8] Bae H , Yoon JS , Choi E , et al. A case of leukaemia cutis in a dog with T‐cell chronic lymphocytic leukaemia. Vet Med Sci. 2022;8(3):947‐952. doi:10.1002/vms3.749 35099125 PMC9122407

[9] Gianella P , Avallone G , Bellino C , et al. Primary cutaneous undifferentiated round cell tumor with concurrent polymyositis in a dog. Can Vet J. 2012;53(5):549‐553. 23115370 PMC3327596

[10] Neiman RS , Barcos M , Berard C , et al. Granulocytic sarcoma: a clinicopathologic study of 61 biopsied cases. Cancer. 1981;48(6):1426‐1437. doi:10.1002/1097-0142(19810915)48 7023656

[11] Usher SG , Radford AD , Villiers EJ , Blackwood L . RAS, FLT3, and C‐KIT mutations in immunophenotyped canine leukemias. Exp Hematol. 2009;37(1):65‐77. doi:10.1016/j.exphem.2008.09.005 18977066

[12] Stokol T , Schaefer DM , Shuman M , Belcher N , Dong L . Alkaline phosphatase is a useful cytochemical marker for the diagnosis of acute myelomonocytic and monocytic leukemia in the dog. Vet Clin Pathol. 2015;44(1):79‐93. doi:10.1111/vcp.12227 25546124

[13] Vernau W , Moore PF . An immunophenotypic study of canine leukemias and preliminary assessment of clonality by polymerase chain reaction. Vet Immunol Immunopathol. 1999;69(2–4):145‐164. doi:10.1016/s0165-2427(99)00051-3 10507302

[14] Yamamoto JF , Goodman MT . Patterns of leukemia incidence in the United States by subtype and demographic characteristics, 1997‐2002. Cancer Causes Control. 2008;19(4):379‐390. doi:10.1007/s10552-007-9097-2 18064533

[15] Kang YS , Kim HS , Park HJ , et al. Clinical characteristics of 75 patients with leukemia cutis. J Korean Med Sci. 2013;28(4):614‐619. doi:10.3346/jkms.2013.28.4.614 23579733 PMC3617317

[16] Kaddu S , Zenahlik P , Beham‐Schmid C , Kerl H , Cerroni L . Specific cutaneous infiltrates in patients with myelogenous leukemia: a clinicopathologic study of 26 patients with assessment of diagnostic criteria. J Am Acad Dermatol. 1999;40(6 Pt 1):966‐978. doi:10.1016/s0190-9622(99)70086-1 10365929

[17] Wong TY , Suster S , Bouffard D , et al. Histologic spectrum of cutaneous involvement in patients with myelogenous leukemia including the neutrophilic dermatoses. Int J Dermatol. 1995;34(5):323‐329. doi:10.1111/j.1365-4362.1995.tb03612.x 7607792

[18] Cho‐Vega JH , Medeiros LJ , Prieto VG , Vega F . Leukemia cutis. Am J Clin Pathol. 2008;129(1):130‐142. doi:10.1309/WYACYWF6NGM3WBRT 18089498

[19] Boudreaux MK , Blue JT , Durham SK , Vivrette SL . Intravascular leukostasis in a horse with myelomonocytic leukemia. Vet Pathol. 1984;21(5):544‐546. doi:10.1177/030098588402100521 6592873

[20] McKee LC Jr , Collins RD . Intravascular leukocyte thrombi and aggregates as a cause of morbidity and mortality in leukemia. Medicine. 1974;53(6):463‐478. doi:10.1097/00005792-197411000-00006 4530890

[21] Comazzi S , Gelain ME , Bonfanti U , Roccabianca P . Acute megakaryoblastic leukemia in dogs: a report of three cases and review of the literature. J Am Anim Hosp Assoc. 2010;46(5):327‐335. doi:10.5326/0460327 20810553

[22] Zhang X , Wang LP , Ziober A , et al. Ionized calcium binding adaptor molecule 1 (IBA1). Am J Clin Pathol. 2021;156(1):86‐99. doi:10.1093/ajcp/aqaa209 33582751

[23] Saravanan L , Juneja S . Immunohistochemistry is a more sensitive marker for the detection of myeloperoxidase in acute myeloid leukemia compared with flow cytometry and cytochemistry. Int J Lab Hematol. 2010;32(1 Pt 1):132‐136. doi:10.1111/j.1751-553X.2008.01124.x 19077157

[24] Tasca S , Carli E , Caldin M , Menegazzo L , Furlanello T , Gallego LS . Hematologic abnormalities and flow cytometric immunophenotyping results in dogs with hematopoietic neoplasia: 210 cases (2002‐2006). Vet Clin Pathol. 2009;38(1):2‐12. doi:10.1111/j.1939-165X.2008.00099.x 19171020

[25] Agis H , Weltermann A , Fonatsch C , et al. A comparative study on demographic, hematological, and cytogenetic findings and prognosis in acute myeloid leukemia with and without leukemia cutis. Ann Hematol. 2002;81(2):90‐95. doi:10.1007/s00277-001-0412-9 11907789

